# Supplementary material for: Systematic investigation of inadequate food access at a large southeastern land grant university
Source: PLoS One. 2024 Mar 6;19(3):e0298041. doi: 10.1371/journal.pone.0298041 (PMC10917268; doi:10.1371/journal.pone.0298041)
Supplement: S1 Table — This table presents the USDA food security metric (i.e., high versus low food security) for each of the main variables included in the study. (DOCX) [file pone.0298041.s001.docx]

**S1 Table: Food Security by Selected Indicators**.

This table presents the USDA food security metric (i.e., high versus low food security) for each of the main variables included in the study.

| **Variable** | **Total Sample Response**  **N (%)** | **High Food Security**  **(% of row)** | **Low Food Security**  **(% of row)** | **P(χ2)** |
| --- | --- | --- | --- | --- |
| **Food Security Status (Q13 to Q16)** | **2116** |  |  |  |
| *High Food Security* | *1777 (84%)* |  |  |  |
| *Low Food Security* | *339 (16%)* |  |  |  |
| **Race/Ethnicity (Select Multiple) (Q57)** | **2096** |  |  |  |
| *American Indian or Alaskan Native* | *30 (1.43%)* | 73.3% | 26.7% | 0.126 |
| *Asian* | *429 (20.5%)* | 84.4% | 15.6% | 0.851 |
| *Black or African-American* | *121 (5.8%)* | 68.6% | 31.4% | <0.001 |
| *Caribbean* | *15 (0.7%)* | 73.3% | 26.7% | 0.277 |
| *Hispanic or Latino* | *151 (7.2%)* | 78.1% | 21.9% | 0.056 |
| *Middle Eastern or North African* | *67 (3.2%)* | 79.1% | 20.9% | 0.350 |
| *Native Hawaiian or Pacific Islander* | *11 (0.5%)* | 63.6% | 36.4% | 0.089 |
| *Sub-Saharan African* | *13 (0.6%)* | 53.8% | 46.2% | 0.009 |
| *White or Caucasian* | *1473 (70.3%)* | 85.5% | 14.5% | 0.003 |
| *Self-identify* | *21 (1.0%)* | 71.4% | 28.6% | 0.142 |
| *Identify as multiple* | *208 (9.9%)* | 78.8% | 21.2% | 0.043 |
| **Gender Identity (Select Multiple) (Q62)** | **2097** |  |  |  |
| *Feminine* | *1150 (54.8%)* | 84.2% | 15.8% | 0.782 |
| *Masculine* | *863 (41.2%)* | 84.5% | 15.5% | 0.613 |
| *Self-identify* | *25 (1.2%)* | 76% | 24% | 0.388 |
| *Gender non-conforming* | *38 (1.8%)* | 73.7% | 26.3% | 0.130 |
| *Questioning* | *20 (1%)* | 70% | 30% | 0.118 |
| *Trans/Transgender* | *12 (0.6%)* | 83.3% | 16.7% | 1.000 |
| *Prefer not to say* | *39 (1.9%)* | 79.5% | 20.5% | 0.588 |
| **Sexual Orientation (Select Multiple) (Q63)** | **2073** |  |  |  |
| *Bisexual* | *246 (11.9%)* | 73.6% | 26.4% | <0.001 |
| *Gay* | *47 (2.3%)* | 78.7% | 21.3% | 0.451 |
| *Heterosexual* | *1614 (77.9%)* | 86.2% | 13.8% | <0.001 |
| *Lesbian* | *35 (1.7%)* | 77.1% | 22.9% | 0.398 |
| *Queer* | *56 (2.7%)* | 73.2% | 26.8% | 0.046 |
| *Questioning* | *45 (2.2%)* | 73.3% | 26.7% | 0.085 |
| *Self-identify* | *35 (1.7%)* | 80% | 20% | 0.702 |
| *Prefer not to say* | *70 (3.4%)* | 75.7% | 24.3% | 0.089 |
| **Level of Education (Q9)** | **2112** |  |  | 0.586 |
| *Undergraduate* | *1343 (63.6%)* | 84% | 16% |  |
| *Graduate* | *738 (34.9%)* | 77.4% | 22.6% |  |
| *Professional* | *31 (1.5%)* | 84.1% | 15.9% |  |
| **First Generation Student (Q50)** | **2058** |  |  | <0.001 |
| *Yes* | 416 (20.2%) | 74.5% | 25.5% |  |
| *No* | 1642 (79.8%) | 86.5% | 13.5% |  |
| **Undergraduate Transfer Student (Q11)** | **1343** |  |  | 0.001 |
| *Yes – 2-year community college* | *111 (8.3%)* | 73% | 27% |  |
| *Yes – 4-year college* | *49 (3.7%)* | 77.6% | 22.4% |  |
| *No* | *1183 (88.1%)* | 85.5% | 14.5% |  |
| **Undergraduate Academic Level (Q10)** | **1339** |  |  | 0.038 |
| *Freshman* | *327 (24.5%)* | 87.5% | 12.5% |  |
| *Sophomore* | *330 (24.6%)* | 85.8% | 14.2% |  |
| *Junior* | *328 (24.5%)* | 82.3% | 17.7% |  |
| *Senior* | *303 (22.6%)* | 82.8% | 17.2% |  |
| *5^th^ Year Senior* | *50 (3.7%)* | 72% | 28% |  |
| **Graduate Academic Level (Q12)** | **759** |  |  | 0.318 |
| *Masters* | *319 (42%)* | 85.3% | 14.7% |  |
| *Doctoral* | *440 (58%)* | 82.3% | 17.7% |  |
| **Residency (Q48)** | **2109** |  |  | 0.003 |
| *In-state* | *1383 (65.6%)* | 83.3% | 16.7% |  |
| *Out-of-state: Domestic* | *444 (21.1%)* | 88.7% | 11.3% |  |
| *Out-of-state: International* | *282 (13.4%)* | 79.8% | 20.2% |  |
| **Assistantship (Q41)** | **737** |  |  | 0.001 |
| *No – Never previously* | *213 (28.9%)* | 92.5% | 7.5% |  |
| *No – Previously had one* | *27 (3.7%)* | 88.9% | 11.1% |  |
| *Yes – 10 hours* | *39 (5.3%)* | 76.9% | 23.1% |  |
| *Yes – 20 hours* | *458 (62.1%)* | 80.3% | 19.7% |  |
| **GPA (Q47)** | **2039** |  |  | <0.001 |
| *< 2.0* | *10 (0.5%)* | 60% | 40% |  |
| *2.00 – 2.50* | *58 (2.8%)* | 67.2% | 32.8% |  |
| *2.51 – 3.00* | *185 (9.1%)* | 71.4% | 28.6% |  |
| *3.01 – 3.50* | *517 (25.4%)* | 86.1% | 13.9% |  |
| *3.51 – 4.00* | *1195 (58.6%)* | 86.6% | 13.4% |  |
| *No GPA Yet* | *74 (3.6%)* | 81.1% | 18.9% |  |
| **Disability (Q58)** | **2110** |  |  | <0.001 |
| *Yes* | *159 (7.5%)* | 71.1% | 28.9% |  |
| *No* | *1785 (84.6%)* | 86.3% | 13.7% |  |
| *Maybe* | *126 (6%)* | 72.2% | 27.8% |  |
| *Prefer not to say* | *40 (1.9%)* | 70% | 30% |  |
| **Does your disability affect your access to food (Q59)** | **262** |  |  | 0.003 |
| *Yes* | *38 (14.5%)* | 52.6% | 47.4% |  |
| *No* | *224 (85.5%)* | 77.2% | 22.8% |  |
| **Are you building up debt to pay for food? (Q21)** | **2034** |  |  | <0.001 |
| *Yes* | *273 (13.4%)* | 53.5% | 46.5% |  |
| *No* | *1761 (86.6%)* | 89% | 11% |  |
| **In the last 12 months, have you spent less on other budget items so that you can afford food? (Q28)** | **1864** |  |  | <0.001 |
| *Yes* | *528 (28.3%)* | 54.4% | 45.6% |  |
| *No* | *1336 (71.7%)* | 96.6% | 3.4% |  |
| **Do you currently have a dining plan? (Q22)** | **2113** |  |  | <0.001 |
| *No* | *1306 (61.8%)* | 83.6% | 16.4% |  |
| *No – I wanted one but wasn’t able to purchase one* | *200 (9.5%)* | 69.5% | 30.5% |  |
| *Commuter Cash Plan* | *16 (0.8%)* | 100% |  |  |
| *Dining Dollars* | *118 (5.6%)* | 91.5% | 8.5% |  |
| *Minor Flex Plan* | *92 (4.4%)* | 90.2% | 9.8% |  |
| *Major Flex Plan* | *209 (9.9%)* | 85.6% | 14.4% |  |
| *Mega Flex Plan* | *97 (4.6%)* | 92.8% | 7.2% |  |
| *Premium Flex Plan* | *75 (3.6%)* | 89.3% | 10.7% |  |
| **Do you have easy access to nutritious meals 1 hour before and/or after class? (Q31)** | **1981** |  |  | <0.001 |
| *Yes* | *1162 (58.7%)* | 94.8% | 5.2% |  |
| *No* | *163 (8.23%)* | 50.9% | 49.1% |  |
| *Depends on the class* | *656 (33.1%)* | 73.6% | 26.4% |  |
| **When your access to food is limited, does it prevent you from enjoying the full university experience? (Q32)** | **817** |  |  | <0.001 |
| *Considerably* | *215 (26.3%)* | 59.1% | 40.9% |  |
| *Greatly* | *87 (10.6%)* | 57.5% | 42.5% |  |
| *Moderately* | *228 (27.9%)* | 65.8% | 34.2% |  |
| *Slightly* | *187 (22.9%)* | 80.7% | 19.3% |  |
| *Not at all* | *100 (12.2%)* | 87% | 13% |  |
| **Living arrangement (Q33)** | *2097* |  |  | 0.061 |
| *On campus* | *394 (18.8%)* | 88.3% | 11.7% |  |
| *Off-campus with parents/guardians* | *133 (6.34%)* | 87.2% | 12.8% |  |
| *Off-campus with spouse/partner/dependents* | *303 (14.4%)* | 84.2% | 15.8% |  |
| *Off-campus by myself* | *261 (12.4%)* | 82.8% | 17.2% |  |
| *Off-campus with roommates* | *1006 (48%)* | 82.3% | 17.7% |  |
| **Living condition** **(Q34)** | *2110* |  |  | <0.001 |
| *Steady place to live* | *1894 (90.6%)* | 88.3% | 11.7% |  |
| *Steady place to live, but am worried about losing it* | *190 (9.1%)* | 45.3% | 54.7% |  |
| *Temporary living/unstable living space* | *6 (0.3%)* | 33.3% | 66.7% |  |
| *No temporary living space* | *1 (0.05%)* |  | 100% |  |
| **Change of the living situation in the last 6 months (Q35)** | **2106** |  |  | <0.001 |
| *Better* | *127 (6%)* | 81.1% | 18.9% |  |
| *Same* | *1749 (83%)* | 86.8% | 13.2% |  |
| *Worse* | *79 (3.8%)* | 68.4% | 31.6% |  |
| *Varies* | *151 (7.2%)* | 63.6% | 36.4% |  |
| **In the past week, I have been depressed (Q66)** | **2105** |  |  | <0.001 |
| *Describes me extremely well* | *245 (11.6%)* | 68.6% | 31.4% |  |
| *Describes me moderately well* | *336 (16%)* | 79.2% | 20.8% |  |
| *Describes me slightly well* | *536 (25.5%)* | 87.9% | 12.1% |  |
| *Describes me very well* | *232 (11%)* | 70.7% | 29.3% |  |
| *Does not describe me* | *756 (35.9%)* | 92.6% | 7.41% |  |
| **In the past week, I have been nervous or worried (Q67)** | **2104** |  |  | <0.001 |
| *Describes me extremely well* | *487 (23.1%)* | 71.7% | 28.3% |  |
| *Describes me very well* | *423 (20.1%)* | 79.7% | 20.3% |  |
| *Describes me moderately well* | *443 (21.1%)* | 88% | 12% |  |
| *Describes me slightly well* | *509 (24.2%)* | 91.6% | 8.4% |  |
| *Does not describe me* | *242 (11.5%)* | 93% | 7% |  |
| **In the past week, when I thought of the future, I was afraid (Q68)** | **2104** |  |  | <0.001 |
| *Not at all afraid* | *367 (17.4%)* | 93.7% | 6.3% |  |
| *A little afraid* | *638 (30.3%)* | 90.6% | 9.4% |  |
| *Somewhat afraid* | *663 (31.5%)* | 81% | 19% |  |
| *Terrified* | *137 (6.5%)* | 66.4% | 33.6% |  |
| *Very afraid* | *299 (14.2%)* | 72.9% | 27.1% |  |
| **In the past week, I have felt that I have no control over my life (Q69)** | **2102** |  |  | <0.001 |
| *Complete control over my life* | *150 (7.1%)* | 96.7% | 3.3% |  |
| *A lot of control over my life* | *660 (31.4%)* | 93.5% | 6.5% |  |
| *Some control over my life* | *816 (38.8%)* | 82% | 18% |  |
| *A little control over my life* | *365 (17.4%)* | 73.2% | 26.8% |  |
| *No control over my life* | *111 (5.3%)* | 61.3% | 38.7% |  |
| **Considering all parts of my life – physical, emotional, social, spiritual, and financial – over the last week the quality of my life has been … (Q70)** | **2103** |  |  | <0.001 |
| *Excellent* | *222 (10.6%)* | 96.4% | 3.6% |  |
| *Good* | *688 (32.7%)* | 93.2% | 6.8% |  |
| *Average* | *806 (38.3%)* | 82.3% | 17.7% |  |
| *Poor* | *333 (15.8%)* | 68.2% | 31.8% |  |
| *Terrible* | *54 (2.3%)* | 40.7% | 59.3% |  |
| **Weight change in the last 12 months (Q53)** | **2109** |  |  | <0.001 |
| *No* | *824 (39.1%)* | 89.2% | 10.8% |  |
| *Yes – Gained Weight* | *755 (35.8%)* | 85.6% | 14.4% |  |
| *Yes – Lost Weight* | *485 (23.0%)* | 74% | 26% |  |
| *Prefer not to say* | *45 (2.1%)* | 73.3% | 26.7% |  |
| **Health Insurance (Q55)** | **2112** |  |  | <0.001 |
| *Yes* | *2000 (94.7%)* | 84.9% | 15.1% |  |
| *No* | *67 (3.2%)* | 62.7% | 37.3% |  |
| *Not sure* | *45 (2.1%)* | 77.8% | 22.2% |  |
| **How has the COVID-19 pandemic affected you? (Q65) (Select multiple)** | **2027** |  |  |  |
| *My ability to engage has not been significantly impacted* | *514 (25.4%)* | 86.4% | 13.6% | 0.073 |
| *My mental health has been negatively impacted* | *1458 (71.9%)* | 81.1% | 18.9% | <0.001 |
| *My physical health has been negatively impacted* | *957 (47.2%)* | 76.3% | 23.7% | <0.001 |
| *I am not exercising as much* | *1237 (61.0%)* | 81% | 19% | <0.001 |
| *I have lost a job* | *202 (10%)* | 62.9% | 37.1% | <0.001 |
| *I have lost access to my regular transportation* | *66 (3.3%)* | 59.1% | 40.9% | <0.001 |
| *I have lost access to my regular childcare* | *22 (1.1%)* | 63.6% | 36.4% | 0.015 |
| *My disposable income has increased* | *143 (7.1%)* | 91.6% | 8.4% | 0.012 |
| *My disposable income has decreased* | *470 (23.2%)* | 64.3% | 35.7% | <0.001 |
| *My housing situation has worsened* | *145 (7.2%)* | 58.6% | 41.4% | <0.001 |
