# Supplementary material for: Systematic investigation of inadequate food access at a large southeastern land grant university
Source: PLoS One. 2024 Mar 6;19(3):e0298041. doi: 10.1371/journal.pone.0298041 (PMC10917268; doi:10.1371/journal.pone.0298041)
Supplement: Attachment — Submitted filename: Response to Reviewers.docx [file pone.0298041.s002.docx]

**November 12, 2023**

**Response to Reviewers**

**Journal requirements**:

When submitting your revision, we need you to address these additional requirements.

1. Please ensure that your manuscript meets PLOS ONE's style requirements, including those for file naming. The PLOS ONE style templates can be found at

<https://journals.plos.org/plosone/s/file?id=wjVg/PLOSOne_formatting_sample_main_body.pdf> and

<https://journals.plos.org/plosone/s/file?id=ba62/PLOSOne_formatting_sample_title_authors_affiliations.pdf>

- **RESPONSE**: Thank you for providing these guidelines. The manuscript has been reformatted so that it complies with PLOS ONE’s style requirements.

2. Please provide additional details regarding participant consent. In the ethics statement in the Methods and online submission information, please ensure that you have specified what type you obtained (for instance, written or verbal, and if verbal, how it was documented and witnessed). If your study included minors, state whether you obtained consent from parents or guardians. If the need for consent was waived by the ethics committee, please include this information.

- **RESPONSE**: As indicated in the methods section, the survey targeted undergraduate and graduate students who were enrolled either as part- or full-time students and aged over 18. Students who did not meet these criteria were excluded. We now explain that consent was obtained at the start of the online survey once the participant had read the consent script and had selected “yes” to a question asking whether they agreed to participate in the study.

3. Please note that PLOS ONE has specific guidelines on code sharing for submissions in which author-generated code underpins the findings in the manuscript. In these cases, all author-generated code must be made available without restrictions upon publication of the work. Please review our guidelines at <https://journals.plos.org/plosone/s/materials-and-software-sharing#loc-sharing-code> and ensure that your code is shared in a way that follows best practice and facilitates reproducibility and reuse.

- **RESPONSE**:  We have included our R code along with the research protocol, survey instrument, and cleaned data set on our OSF site. This link will be made public if the paper is accepted: <https://osf.io/bnuqv/?view_only=977d9eefa53d4e61b0ce61eada8cb048>

4. Thank you for stating the following in the Funding Section of your manuscript:

“The initial research that made this study possible was supported by funding from the Virginia Tech Center for International Research, Education, and Development (CIRED), the office of Outreach and International Affairs (OIA), and the College of Science.”

We note that you have provided funding information that is not currently declared in your Funding Statement. However, funding information should not appear in the Acknowledgments section or other areas of your manuscript. We will only publish funding information present in the Funding Statement section of the online submission form.

Please remove any funding-related text from the manuscript and let us know how you would like to update your Funding Statement. Currently, your Funding Statement reads as follows:

“The funders had no role in study design, data collection and analysis, decision to publish, or preparation of the manuscript.”

Please include your amended statements within your cover letter; we will change the online submission form on your behalf.

- **RESPONSE**: We have removed the funding statement from the manuscript as requested and will revise the Funding Statement as follows: “The initial research that made this study possible was supported by funding from the Virginia Tech Center for International Research, Education, and Development (CIRED), the office of Outreach and International Affairs (OIA), and the College of Science. These entities had no role in the study design, data collection and analysis, decision to publish, or preparation of the manuscript.”

5. We note that you have stated that you will provide repository information for your data at acceptance. Should your manuscript be accepted for publication, we will hold it until you provide the relevant accession numbers or DOIs necessary to access your data. If you wish to make changes to your Data Availability statement, please describe these changes in your cover letter and we will update your Data Availability statement to reflect the information you provide.

- **RESPONSE**: If the manuscript is accepted for publication, all of the data used in the analysis will be publicly accessible via our OSF site: <https://osf.io/bnuqv/?view_only=977d9eefa53d4e61b0ce61eada8cb048>. We can provide the DOI for the data set as soon as the OSF site is made public.

6. Please include captions for your Supporting Information files at the end of your manuscript, and update any in-text citations to match accordingly. Please see our Supporting Information guidelines for more information: <http://journals.plos.org/plosone/s/supporting-information>.

- **RESPONSE**: The Appendix has been renamed “S1 Table.” All in-text citations have been changed to “S1 Table,” and the following caption has been added at the end of the paper: “S1 Table: Food Security by Selected Indicators. This table presents the USDA food security metric (i.e., high versus low food security) for each of the main variables included in the study.”

**Additional Editor Comments**:

The authors are commended for their attempt to add to the extant literature on the topic of food accessibility in institutions of higher education.  The utilization of a mixed methods approach to capture both quantitative and qualitative data on the topic is laudable.  However, as pointed out in the review, there are several areas in need of revision.

In the Introduction the inclusion of supporting references is needed.  For instance, on lines 39 – 44 the authors fail to provide empirical support for statements regarding evidence of the role of food insecurity on student performance and quality of life.

- **RESPONSE**: We have added the needed citations to the statements being made in this paragraph.

On line 49 it is unclear as to what “rite of passage’ refers to.  As Reviewer 1 queries “Is this referring to experiencing challenges accessing food as a “rite of passage.” This statement is vague and should be clarified.

- **RESPONSE**: We have added “lack of access” to the statement about “safe, affordable, nutritious, and culturally relevant foods” to clarify what “rite of passage” is referring to. Experiencing a lack of access to food is considered by some to be a part of the university experience. Our research team encountered this belief when sharing the results from this study at our institution, with some arguing that their poor access to food was a part of their personal development as an undergraduate/graduate student. We decided to include the “rite of passage” statement to challenge the notion that food insecurity is a ‘normal’ part of the college experience.

The authors should also check lines 55 – 56 (i.e., “There are is..”) and line 67 (i.e., Recent research explores…) for grammatical errors.

- **RESPONSE**: Thank you for catching these errors. They have been corrected.

More information is needed on the 10-item USDA Household Food Security Instrument.  This is the instrument used to assess food security and more details are needed on how the measure is used and how it has been validated if not on a college student population as mentioned.  This measure assesses the primary construct of interest and should be fully described.

- **RESPONSE**: We have provided additional information about the 10-item USDA instrument, which includes references that discuss its evolution and use since its creation in 1995. We also cite the 2006 National Research Council (NRC) study, which is considered one of the most rigorous studies of the USDA instrument. Many of the recommendations in the NCR study were subsequently incorporated into the instrument by the USDA. The instrument is widely used in various national surveys, including the Current Population Survey (CPS) and is considered to be a valid and reliable instrument for measuring the food security of a population.

In the Methods section on line 132 it states that “The word household was removed from the module” however, in Table 1 the word “household” is included.  Please clarify.  It is also unclear if Question 1 in the table is a screener question.  It appears to be a screener question and if so, this should be stated.

- **RESPONSE**: Thank you for catching this. We have revised Table 1 so that it now matches the survey text. We also added the word “Screener” to the first row of the table to indicate this text was the screener question. While the word household was removed from the screener question, it was retained, with qualifications, in the instrument. These qualifications were added to try and remove any uncertainty about what was meant by the term household. We have added additional text to the methods section to clarify the adjustments that were made.

A description of all measures assessed should be included in this section.  For example, demographic questions pertaining to race/ethnicity and income are not included.  There is also no mention of a measure or items that assess mental health.

- **RESPONSE**: In the Methods sections we have added a description of the additional measures included in the survey and have cited the instruments/surveys that informed the mental health and housing questions.

On line 167 the qualitative method used to analyze open-ended responses is provided.  More information on this method and a reference is needed.  What prompts or questions were provided to participants?  Was inter-rater reliability assessed? Was qualitative software used to identify themes?  On line 165 it is mentioned that “coding was conducted using Word”, this is vague.

- **RESPONSE**: We have added additional detail on the qualitative analysis approach that we used. Following the method of Thomas and Harden (2008), we opted to engage in a group discussion of overlaps and divergences of qualitative codes rather than merely assessing the extent of consensus.
- Thomas, J., Harden, A. Methods for the thematic synthesis of qualitative research in systematic reviews. *BMC Med Res Methodol 8*, 45 (2008). <https://doi.org/10.1186/1471-2288-8-45>

The Results section could be strengthened if more details about the sample and measures are provided.  Without more details (e.g., race/ethnicity) it is difficult to interpret the findings. What percentage of the sample is Black or African American?  Has sample size been adjusted for small n’s?

- **RESPONSE**: We have revised the S1 Table so that it now includes additional variables on race/ethnicity, gender identity, sexual orientation, and COVID-19. As mentioned in the methods section, a permutation chi square test was conducted when the sample size in any individual group was smaller than or equal to 5.

The figures are blurred and difficult to see.  Be sure to follow submission guidelines for submitting figures and graphs.

- **RESPONSE**: We selected an appropriate R package to revise/recreate all of the figures in the manuscript. Since we needed to adjust how several variables were included in the analysis, a number of the findings have been revised. All of the figures have been processed using the PLOS ONE Preflight Analysis and Conversion Engine (PACE) digital diagnostic tool.

The authors provide recommendations at the end of each result presented.  It may be best to just present the findings in the Results section and in the Discussion section provide a summary of the findings with recommendations.

In the Discussion section, qualitative responses are included.  It is more appropriate to present these findings in the Results section as this is a mixed-methods study.  As cited earlier, more details are needed on the qualitative component of the study and themes that emerged.

- **RESPONSE**: We have revised the paper so that the qualitative results are now presented in the Results section and the recommendations are included in the Discussion section.

Other comments relate to other areas the authors should consider.  As the reviewer suggests, provide potential explanations to why the prevalence of food insecurity was lower in this study than in others. Knowing more about the university and campus locations students were recruited from may assist with this.

- **RESPONSE**: We have undertaken additional analysis on a survey question related to COVID-19 that we believe helps enrich our interpretation of the findings. Please see Fig 9, the related data in S1 Table, and the revised text below that speaks to the lower level of food insecurity found in this study.

“The percentage of survey respondents found to have a low food security status (16%) is lower than what is typically found in studies at other comparable institutions of higher education (9,30,38). Following an initial survey of food access and security at the university (62), a food security taskforce was convened in 2020 that identified a series of barriers students face when accessing food and presented a range of recommendations and actions to address them (63). The most significant action taken was the creation of a free food service that could provide over 100 students a week with access to seasonal produce and other food items. This service began as a pilot program in 2020 and was formally launched in 2022 with additional donor support. In parallel with this, the university’s Dean of Students Office dedicated a portion of its emergency assistance fund to support students with food access needs via one-time grants of typically between $600-$800.

Since 2019, the university has also significantly enhanced its communication of these support services, which also includes information about off-campus resources such as a local food pantry that is available to all students. In addition to the university’s actions, the Coronavirus Aid, Relief, and Economic Security Act (CARES Act) provided three rounds of direct relief payments to eligible adults and qualifying children, two of which were released during the year prior to the survey.

While it is not possible to make a direct causal connection between the higher level of food security and the actions taken by the university or funding that may have been received via the CARES Act, written feedback from participants indicates that a number of students benefitted significantly from the university support they received. The higher level of food security could also be related to a self-selection bias due to COVID stressors. For example, students experiencing a decline in mental and physical health may not have had the capacity to complete the survey.”

How many of these students live on campus versus off campus?  How many live with their family or a roommate as this too may aid in data interpretation.

- **RESPONSE**: The S1 Table provides the following data on where students are living and how this corresponds with food security. As can be seen below, 394 students lived on campus, 142 were living off-campus with their parents/guardians, 304 were living off-campus with their spouse/partner/dependents, and 1,271 were living off-campus by themselves or with roommates. Students living off campus with roommates were found to be 1.53 times more likely to have a low food security status when compared with students living on campus (see Fig 6).

| **Variable** | **Total Sample Responded**  **N (%)** | **High Food Security**  **% of row** | **Low Food Security**  **% of row** | **P(χ2)** |
| --- | --- | --- | --- | --- |
| **Living arrangement (Q33)** | *2097* |  |  | 0.061 |
| *On campus* | *394 (18.8%)* | 88.3% | 11.7% |  |
| *Off-campus with parents/guardians* | *133 (6.34%)* | 87.2% | 12.8% |  |
| *Off-campus with spouse/partner/dependents* | *303 (14.4%)* | 84.2% | 15.8% |  |
| *Off-campus by myself* | *261 (12.4%)* | 82.8% | 17.2% |  |
| *Off-campus with roommates* | *1006 (48%)* | 82.3% | 17.7% |  |

Additionally, there seems to be vacillation between the term “low food security” and “food insecurity”.  Are the authors suggesting a difference between the two terms?  Are they the same?  If so, it may be easier and more efficient to use one or the other.  An operational definition of food insecurity might help.

- **RESPONSE**: In an effort to minimize any confusion in our use of terms, we have removed reference to high/marginal and low/very low in the Results, Discussion and Recommendations, and Conclusion sections of manuscript, and now only use high and low food security. We have also made changes where possible to ensure food insecurity is used appropriately.

Given that the study was conducted in April 2021 during the Covid-19 pandemic, this should also be considered as a potential factor influencing findings.  In addition to participant recall, this too may be a potential confound and limitation.

- **RESPONSE**: Please see the previous response that discusses the new analysis we have undertaken on a COVID-19-related question.

Overall, the topic is very interesting and has the potential to address factors associated with food insecurity on a college campus. As written, the paper is not accepted for publication but with significant revisions will be reconsidered.

- **RESPONSE**: We have completely revised the manuscript in response to the feedback provided.

**Reviewers' comments**:

Reviewer's Responses to Questions

Reviewer #1: Introduction
Lines 39-44: Add references

- **RESPONSE**:  We have added the needed citations to the statements being made in this paragraph.

Lines 48-50: The wording of this sentence is a little unclear. It sounds like it’s saying that access to food is considered a “rite of passage.” Is this referring to experiencing challenges accessing food as a “rite of passage.”

- **RESPONSE**: We have edited this sentence to clarify the rite of passage statement. Please see the prior comment for additional information.

Lines 55-56: This is the first time the 10-item USDA Household Food Security Survey instrument is mentioned. It would be helpful to briefly mention what this is and that it’s a commonly used measure for assessing food security status in the US.

- **RESPONSE**: We have added additional information on the 10-item USDA instrument, including that it is a module in the annual US Current Population Survey (CPS). Please see the prior comment for additional information.

Lines 58-60: How does it allow for comparable results?

- **RESPONSE**: Since the majority of food security research at institutions of higher education are based on the USDA Household Food Security Survey instrument, using this instrument means that the research can be compared with these studies. We have revised the text to indicate that the 10-item USDA instrument has “been found to perform better than the 6-item version with regards to measuring food security among the college student population.”

Methods
Line 132: This says the word household was removed from the module, but the first question in table 1 uses household.

- **RESPONSE**: We have revised the text to indicate how the screener and subsequent questions of the 10-item USDA instrument were edited to ensure that students would be able to interpret the questions more clearly. Please see the prior comment for additional information.

Since “high” and “low” are two of four USDA categories, I suggest referring to the two groups as food secure and food insecure as commonly done in other studies. The results section is confusing because it’s unclear if you’re referring to just the low food security group or low/very low combined. There is also inconsistency in the results section as to how it’s being referred to.
The methods section only describes the questions used to assess food security status. Briefly describe the other questions on the survey. There doesn’t need to be as much detail as for food security but some mention of other information being collected would be helpful.

- **RESPONSE**: As mentioned above, in an effort to minimize any confusion in our use of terms, we have removed reference to high/marginal and low/very low in the manuscript, and now only use high and low food security. We have also made changes where possible to ensure food insecurity is used appropriately.

Lines 159-160: Was R used just for the odds ratios or also for the chi-square and Fisher’s exact tests described in the next paragraph. If it was used for all, you may want to move that sentence to the beginning or end of the information on statistical analyses to help clarify.

- **RESPONSE**: R was used for the odds ratios and the chi-square tests. The sentence was moved to the beginning of the paragraph.

Lines 167-169: How many coders were there and were responses double-coded or coded by a single coder?

- **RESPONSE**: Additional clarification has been provided in the Methods section. There were three coders who conducted simultaneous coding of the qualitative responses. A group discussion of overlaps and divergences was then conducted following the method of Thomas and Harden (2008).

Results
Lines 175 and 176: Be consistent in use of commas in numbers.

- **RESPONSE**: The manuscript has been edited to ensure we present numerical data in a consistent way.

Line 176: You may want to provide a little context about the university in the methods section as this is the first time having a main campus was mentioned. Many universities only have a single campus, so you may want to mention how many campuses there were in the methods and if the majority of students attended the main campus.

- **RESPONSE**: Table 3 provides descriptive data on sample and survey population. The majority (96.5%) of our students are based at the main campus in Blacksburg, and 93.5% of our respondents were based at this campus. We have added the following sentence to the methods sections that talks about our campuses: “The majority of the 2,116 respondents (94%) were enrolled at the university’s main campus in Blacksburg (Table 3). The university also has a presence in northern Virginia with several facilities located around the greater Washington, D.C., metro area, and has a small presence in Roanoke and Richmond, Virginia.”

Table 4: Rather than saying “by Selected Demographic Characteristics” you may want to say something like student classification.

- **RESPONSE**: We have revised the name of the table so that it better describes the data that is being presented. The new label for Table 3 is “Comparison of the Sample and Survey Population.”

Line 220: The tables and figure seem to indicate that the question on being a first generation college student was a yes/no question and that data wasn’t collected to determine if a student was a second generation college student. Not all students who are not first generation college students are second generation – some may have grandparents, great grandparents, etc. who also attended college.

- **RESPONSE**: This is a great point and is something that we will endeavor to be more precise about in future research. The survey only asked respondents if they were a first-generation student. This question was connected with the First-Generation Student Support office at our university, which reaches out and communicates with students who meet the following definition: “At [... name of institution], a student is identified as a first-generation college student if neither parent/guardian has earned a bachelor’s degree at a four-year college or university.” Thus, respondents should know if they are a first-generation student based on this definition and the services they can access. Our analysis compares students who consider themselves to be first-generation students, with those who do not consider themselves to be first-generation students.

Lines 235-236: I’m not sure that saying there is an important difference between the groups is the best way to reference that there was a large difference in the odds ratios. You may want to take out this sentence or reword to focus more on there being a difference in size of odds rations.

- **RESPONSE**: Agree. We have removed this statement.

Discussion
Line 387: The way this is worded sounds like 7% of those who lived with their parents. Is this trying to say that 7% of students in the sample lived with their parents?

- **RESPONSE**: We have revised this text to make it clear that around 6% of the sample were living with their parents.

Lines 475-476: You may want to briefly explain what work study is.

- **RESPONSE**: Additional text has been provided to explain how the Federal Work-Study (FWS) program functions.

Lines 480-515: There are many reasons why the prevalence of food insecurity was lower in this study than some others. You may want to mention some potential other reasons as well. This is a great example of a program the university is offering, but will a program reaching up to 115 students from a university of over 35,000 students likely be having a major impact on the prevalence of food insecurity on the campus?

- **RESPONSE**: Please see the previous response that discusses the new analysis we have undertaken on a COVID-19-related question.

Line 559: For future studies, you may want to keep in mind that there is both a 12-month and 30-day recall period for the 10-item US Household Food Security Survey Module.
Limitations

- **RESPONSE**: We have provided additional text in the introduction and methodology section about the 12-month and 30-day recall versions of the USDA 10-item Household Food Security Survey Module. This text indicates that the 30-day recall version is intended to study specific programs. We have added some additional text to the Limitations section which states that neither the 12-month or 30-day recall options may be ideal and that a revised instrument should be tested that does not refer to households and uses a recall period that aligns with the academic calendar.

Another limitation to mention is external generalizability as this study included students from a single university

- **RESPONSE**: We have added the following sentence to the Limitations section: “Finally, since this study focuses on a single university, care should be taken when trying to generalize the findings to other institutions of higher education.”

While revising your submission, please upload your figure files to the Preflight Analysis and Conversion Engine (PACE) digital diagnostic tool, <https://pacev2.apexcovantage.com/>. PACE helps ensure that figures meet PLOS requirements. To use PACE, you must first register as a user. Registration is free. Then, login and navigate to the UPLOAD tab, where you will find detailed instructions on how to use the tool. If you encounter any issues or have any questions when using PACE, please email PLOS at figures@plos.org. Please note that Supporting Information files do not need this step.

- **RESPONSE**: All of the figures have now been configured using PACE. Since the PLOS ONE PDF Builder makes the figures hard to read, we have included each figure below for your reference.

**Fig 1. Odds Ratio and Confidence Interval for GPA, Disability, and Residency Variables**

**Fig 2. Odds Ratio and Confidence Interval for Undergraduate Academic Level, Transfer, and First-Generation Variables**

**Fig 3. Odds Ratio and Confidence Interval for Graduate Level and Assistantship Variables**

**Fig 4. Odds Ratio and Confidence Interval for Food- and Expenditure-Related Variables**

**Fig 5. Odds Ratio and Confidence Interval for Race/Ethnicity**

**Fig 6. Odds Ratio and Confidence Interval for Housing Variables**

**Fig 7. Odds Ratio and Confidence Interval for Mental Health Variables**

**Fig 8. Odds Ratio and Confidence Interval for Quality of Life, Insurance, and Weight Variables**

**Fig 9. Odds Ratio and Confidence Interval for COVID-related Question**
