# Supplementary material for: Systematic investigation of inadequate food access at a large southeastern land grant university
Source: PLoS One. 2024 Mar 6;19(3):e0298041. doi: 10.1371/journal.pone.0298041 (PMC10917268; doi:10.1371/journal.pone.0298041)
Supplement: Attachment — Submitted filename: Response to Reviewers_Dec_2023.docx [file pone.0298041.s003.docx]

**December 21, 2023**

**Response to Reviewers**

Once again, the authors are commended for addressing the topic of low food security among students in institutions of higher education. The revised manuscript addresses concerns raised in the previous review and is accepted with minor revisions. Most revisions are editorial. Recommended minor revisions are as follows:

- **RESPONSE**: Thank you for reviewing the manuscript again. The updated manuscript addresses each of the comments below.

Introduction: No recommendations provided.

Methods: It is recommended that information about IRB compliance (i.e., The study was undertaken in compliance with the institutional research protocol IRB-21-122) be included after the description of the procedures (i.e., On or after Line 126).

- **RESPONSE**: We have added the following sentence to the paper: “*The study was undertaken in compliance with the institutional research protocol IRB-21-122*.” Further, the research protocol, survey instrument, R code, and cleaned data set have been uploaded to the OSF site. This link will be made public if the paper is accepted: <https://osf.io/bnuqv/?view_only=977d9eefa53d4e61b0ce61eada8cb048>

Results: On line 221, use the term “Approximately” rather than “Around” and drop the word “both”.

- **RESPONSE**: We have made this revision. The sentence now reads: “*Approximately 16% of undergraduate and graduate students can be classified as having low food security (Table 4).*”

• Line 227, delete “that were”.

- **RESPONSE**: We have made this revision. The sentence now reads: “*The socio-demographic and economic characteristics linked to a higher likelihood of experiencing low food security included*:”

• Lines 255 – 257. Was type of disability assessed? Did these students have a physical disability making it possibly more challenging to access food if appropriate resources were not available? This is an interesting finding as it confirms recent research on college students with disabilities and food insecurity (Stott GN, Taetzsch A, Morrell JS. College students with disabilities report higher rates of food insecurity. Disabil Health J. 2023 Oct;16(4):101485. doi: 10.1016/j.dhjo.2023.101485. Epub 2023 Jun 1. PMID: 37353371.). Understanding factors associated with student disability status and food insecurity could be an avenue for future research.

- **RESPONSE**: Thank you for sharing the above article and raising this point. Unfortunately, we did not capture the type of disability a student might have in order to maintain student privacy. If we run the survey again in 2025, we will consider collecting this information to build on the work of Stott et al. (2023). We have revised the text in this section as follows: “*Students who self-assessed that they had a disability were 2.8 times more likely to have a low food security status when compared with students without a disability (Fig 1). The type of disability was not requested in order to maintain student privacy.*” We have also added some new text on the need for research that understands the factors associated with student disability status and food insecurity to the Limitations section (see below).

• Line 313, add the word “to” (i.e., mean students have access to the food they need due to dietary restrictions.)

- **RESPONSE**: We have made this revision. The sentence now reads: “*Further, having a dining plan does not mean students have access to the food they need due to dietary restrictions.*”

• Line 356, remove the word “some” (i.e., but 13% of students were still…)

- **RESPONSE**: We have made this revision. The sentence now reads: “*This group had one of the highest levels of food security (87.2%), but 13% of students were still experiencing food access problems (see S1 Table).*”

• Line 357, use the term “Approximately” rather than “Around”.

- **RESPONSE**: We have made this revision. The sentence now reads: “*Approximately 12% of the students living on campus had a low level of food security, compared with 17% of students living off campus by themselves or with roommates.*”

Discussion and Recommendations: On line 452 it is unclear if the recommendations provided address the preceding section in which it is discussed that first-generation or transfer students, GPA, disability status, and being Black/African-American predicts greater odds of low food security. The recommendations as stated are general and do not specifically address recommendations for these groups. While it is understood that strategies informing “first-year and existing students of the resources available to them” is important, providing specific recommendations or at best mentioning these groups who are potentially at-risk for low food security is important and reinforces the findings.

- **RESPONSE**: We have revised the text as follows to make sure the specific groups of impacted students are mentioned: “*Developing strategies to inform first-year and existing students, especially those at higher risk of food insecurity (i.e., students with a disability, minority students, and low academically performing students), of the resources available to them throughout their studies is recommended. University administrations can work most effectively through student groups, associations, and initiatives that already reach students most at risk of experiencing food security to provide tailored information on available resources.*”

Other comments: Line 593 – Limitations and Direction for Future Research should precede the section on Conclusions. The Conclusions should be listed last.

- **RESPONSE**: The Limitations section now proceeds the Conclusions. We have also added the following sentence that speaks to the need for more research on how students with disabilities could be better served: “*While this research did not capture the type of disability a student might have in order to maintain student privacy, understanding the factors associated with student disability status and food insecurity is an important avenue for future research (64)*.”

Again, the topic is very interesting and has the potential to address factors associated with food insecurity on a college campus. The authors have addressed previous concerns and have provided the recommended revisions. As mentioned, the paper should be accepted for publication with minor revisions.

- **RESPONSE**: Thank you for taking the time to help us further improve the manuscript.
